# Vignettes: an innovative qualitative data collection tool in Medical Education research

**DOI:** 10.1007/s40670-024-02074-0

**Published:** 2024-06-05

**Authors:** Sylvia Joshua Western, Brian McEllistrem, Jane Hislop, Alan Jaap, David Hope

**Affiliations:** College of Medicine & Veterinary Medicine, The University of Edinburgh, Edinburgh, UK

**Keywords:** Vignettes, Qualitative research, Medical education, Data collection

## Abstract

This article describes how to make use of exemplar vignettes in qualitative medial education research. Vignettes are particularly useful in prompting discussion with participants, when using real-life case examples may breach confidentiality. As such, using vignettes allows researchers to gain insight into participants’ thinking in an ethically sensitive way.

Vignettes are written, visual, or oral stimuli portraying realistic events in a focussed manner, purposefully aligned with the research objectives and paradigms to elicit responses from research participants [1]. They have been used in qualitative research to explore physical, social, and mental health–related topics. Although clinical vignettes are widely used in teaching and assessment, vignettes are under-utilised as a research tool in medical education. In this article, we outline the ways in which we found vignettes to be helpful in addressing our research aims prompting a conversation on how they might be used in other medical education research contexts, particularly when working with sensitive issues.

We used vignettes within individual semi-structured interviews, to explore how medical educators interpreted different test-wise behaviours (“*skills and strategies that are not related to the construct being measured on the test but that facilitate an increased test score*”[2]). We opted to use vignettes for the following reasons:

- Akin to clinical vignettes, they enable usage of anonymised and fictionalised version of real-life case studies, protecting the identity and confidentiality of the original individuals [3]. Vignettes retained the essence of the event but potential identifiers or personal information from the original were redacted or anonymized.
- Realistic scenarios support the exploration of sensitive topics which can generate authentic ethical dilemmas. Instead of asking “Have you ever tried to trick your examiner into giving you more marks”? - a question which might cause distress or harm to participants, we could posit a vignette and ask our participants for a third-person perspective. Vignettes therefore promote participants’ psychological safety by providing an alternative non-confronting and safer avenue to discuss value-laden constructs [1].
- When discussing complex ambiguous topics, they provide a focus to help participants orient to the specific matter at hand [3]. Vignettes help define and communicate the context, setting, character, and situation succinctly.

Using an established framework of Skilling & Stylianides [1], we constructed five vignettes portraying a spectrum of test-wise behaviours. We drew on informal conversations with stakeholders, online forums, our professional experience, academic literature, and knowledge of the local context to draft the vignettes. Our aim was to understand how people make meaning, what guided their decisions and reactions to test-wise behaviours. Following feedback from experts and several pilot interviews, we revised the vignettes. As such we found that the process of building vignettes was iterative, collaborative, and continuously evolving.

Using previous case studies employing vignettes for data collection, we reflected on the iterative process of constructing, peer and expert reviewing, piloting, and deploying vignettes to eight participants. Participants were staff and students at Edinburgh Medical School. By contemplating the decision-making pathway that aided vignette construction, studying the reflective notes of the interviewer, thematically analysing interview transcripts, and engaging in an ongoing discussion and feedback loops with our expert and supervisory panel, we identified eight factors making vignettes especially useful:

## Vignettes could uncover biases

By controlling the age, sex, and ethnicity of subjects, we could explore how participants interpreted and reacted to different test-wise behaviours of different students.

## Vignettes allowed for co-creation

Following discussion, participants commented on the realism of the vignettes, allowing for iteration of the vignettes over time.

## Vignettes gave insights into group culture

Vignettes facilitated subjective interpretation of complex situations and allowed for intentional reflection on thoughts and actions.

## Vignettes opened discussion

Participants had the agency to discuss their own attitudes in relation to the vignettes and used them to explore their real-life experiences.

## Vignettes allowed for flexibility

We tailored the frequency and type of vignette based on the participant’s role, and selected vignettes to explore issues under-discussed in previous interviews.

## Vignettes aided pragmatism and openness

Criticising real actions and guidelines can be challenging. Discussing hypothetical vignettes allowed for openness, honesty, and pragmatic answers.

## Vignettes helped confirm validity

Exposing participants to novel vignettes helped the researchers compare their expectations and beliefs to participant views. Participants found the vignettes plausible, which suggested the researchers had a defensible understanding of the topic.

## Vignettes allowed comparison and follow-up

We can compare the interpretation of the same vignette by different individuals in different roles to understand the underlying rationale for their differing perspectives. Follow-up interviews allow for the exploration of changes over time.

**Fig. 1:**
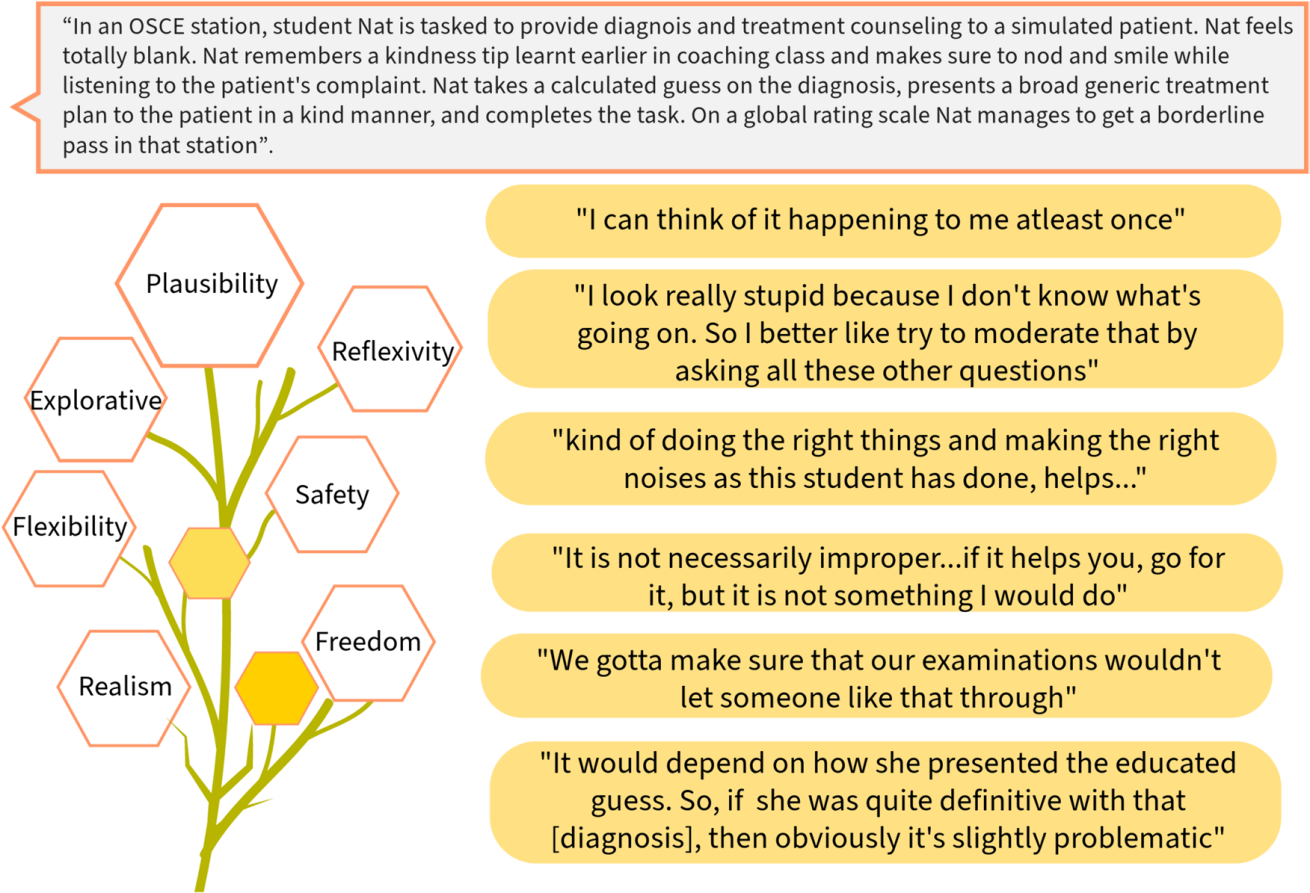
Example vignette with excerpts of participant responses

Figure 1 shows an exemplar vignette with excerpts of participant responses. Rather than ask how they would feel if an exam candidate used false empathy to conceal their lack of content knowledge, we used Nat vignette (in Fig. 1) as a realistic case study to facilitate discussion. The broader themes in the left side of the infographic (Fig. 1) speak to some of the factors identified previously, acting as a teaser facilitating the readers to think through the participant responses. For example, the snippet “I can think of it happening to me at least once” connects to plausibility and realism - the participant thinks that this is a plausible scene in their context, and it seems real to them.

Firstly, a challenge we faced pertained to participant engagement. While all participants found the example vignette (in Fig. 1) both plausible and relatable, the pattern of engagement varied among them. Some used it as a springboard to delve into their own real-life stories, while others found it challenging to reconcile the artificial and hypothetical nature of the vignette. The effectiveness of vignettes hinges on participant engagement. Drawing from our experience and the supporting literature, we found that vignettes must be relatable [3], plausible [3], and situated in context [1]. Participants must be oriented to the vignette method before interview and be given the vignettes at appropriate times during the interview. It is essential when using vignettes to gauge and promote engagement during the interview. Tailored questions and prompts are helpful strategies to promote such engagement. Secondly, we agree that however realistic vignettes are, they are “not real”, therefore participants’ responses to hypothetical vignettes might not perfectly align with their reactions to real-life situations, for instance, considering their underlying motivational relevance to the different contexts - research environment and real-life [3]. Researchers should remain aware of these challenges and interpret their findings with caution [3].

In conclusion, our use of vignettes was an innovative alternative to using high-stakes, confidential real-life case examples in qualitative research. Usage of vignette opens new possibilities in medical education research: they can be used within questionnaire surveys, individual and focus group interviews, or as ethnographic field notes. They offer a versatile approach to allow exploration of high-stakes, sensitive, and ethically contentious issues with participants in a safe way. Therefore, researchers can benefit significantly from applying vignettes in their own research.

## Data Availability

The datasets generated during and/or analysed during the current study are available from the corresponding author on reasonable request.
